# Development and Evaluation of A Novel and Cost-Effective Approach for Low-Cost NO_2_ Sensor Drift Correction

**DOI:** 10.3390/s17081916

**Published:** 2017-08-19

**Authors:** Li Sun, Dane Westerdahl, Zhi Ning

**Affiliations:** 1 School of Energy and Environment, City University of Hong Kong, Tat Chee Avenue, Kowloon, Hong Kong, China; lisun4-c@my.cityu.edu.hk (L.S.); danewest03@gmail.com (D.W.); 2 Guy Carpenter Climate Change Centre, City University of Hong Kong, Tat Chee Avenue, Kowloon, Hong Kong, China

**Keywords:** electrochemical sensor, sensor drift, zero correction

## Abstract

Emerging low-cost gas sensor technologies have received increasing attention in recent years for air quality measurements due to their small size and convenient deployment. However, in the diverse applications these sensors face many technological challenges, including sensor drift over long-term deployment that cannot be easily addressed using mathematical correction algorithms or machine learning methods. This study aims to develop a novel approach to auto-correct the drift of commonly used electrochemical nitrogen dioxide (NO_2_) sensor with comprehensive evaluation of its application. The impact of environmental factors on the NO_2_ electrochemical sensor in low-ppb concentration level measurement was evaluated in laboratory and the temperature and relative humidity correction algorithm was evaluated. An automated zeroing protocol was developed and assessed using a chemical absorbent to remove NO_2_ as a means to perform zero correction in varying ambient conditions. The sensor system was operated in three different environments in which data were compared to a reference NO_2_ analyzer. The results showed that the zero-calibration protocol effectively corrected the observed drift of the sensor output. This technique offers the ability to enhance the performance of low-cost sensor based systems and these findings suggest extension of the approach to improve data quality from sensors measuring other gaseous pollutants in urban air.

## 1. Introduction

Sensors of various types have been introduced for use in air quality monitoring as a means to simplify and reduce the cost of pollution measurement. These are often known as “low-cost sensors” that span a wide range of unit cost (bare sensor cost) from a few USD to one or two thousand USD [1]. There are sensors that are offered for common pollutants, such as ozone, nitrogen dioxide, nitric oxide, carbon monoxide, carbon dioxide, and sulfur dioxide. Many of these sensors have known performance issues that may limit their application. The best known include interactions with other gases, as well as temperature and humidity [2,3]. A less well characterized parameter is long-term stability or drift of the sensor response when continuously operated for periods of weeks, months, or even years [4]. If these factors are not controlled or considered by sensor users, data quality and reliability may be significantly challenged.

Electrochemical sensors are one type of low-cost sensors commonly used to measure constituents of ambient and micro-environmental air. One reason for making such measurements is to learn about human exposures to harmful gases. NO_2_ is one of these pollutants of interest due to health concerns [5,6,7] both in outdoor and indoor environments and is measured by small low-cost gas sensors; commonly by electrochemical cells. It is well known that electrochemical sensors respond differently to a given concentration of their target gas under complex and variable conditions, as well as during periods of environmental transition [8]. Failure to account for these factors may cause reported NO_2_ levels to be inaccurate. Studies have employed various mathematical means to correct the impacts of these factors including developing regression adjustments, such as pattern recognition algorithms [9], artificial neural networks [10], and multiple-input-single-output neural networks, taking observations from comparisons of the sensor response vs. various inputs [11]. Others have used nearby pollutant and environmental monitoring station data for post-observation corrections [8,12]. 

Conventional air monitoring for gaseous pollutants commonly include on-site protocols which serve to assure data quality including periodic challenges with zero gas and span gas of known concentrations. This approach is not commonly employed in the field operations of low-cost sensor systems due to complex setup requirements and high cost. Earlier studies have documented manual protocols for sensor-based systems by observing sensor readings during periods of minimal ambient concentrations is achievable to do the sensor auto-calibration [8]. However, this method is not applicable and practical when sensors are deployed in places where reference concentration data is not available, such as indoor monitoring, exposure assessment, or operations in remote locations.

In this study, we developed a low cost and field-deployable system for sensor signal drift auto-correction, taking NO_2_ as the target gas for demonstration. A working assumption was that electrochemical cells produce a linear response to known concentrations of gas under relatively constant temperature and relative humidity (RH) condition [2,13]. The generation of the NO_2_ free gas was based on the utilization of a chemical absorbent [14], which is a spherical and porous pellet made of activated alumina, binders, and sodium permanganate. The impacts of the absorbent on gas stream RH and the effectiveness over a wide range of ambient NO_2_ concentrations were extensively evaluated. Both mathematical correction algorithm and physical humidity conditioning using a Nafion tube were applied in the system to assess improvements on sensor performance under controlled conditions. A comprehensive field evaluation in different environments was also carried out to validate the effectiveness of the developed system in sensor drift correction operated in ambient air in Hong Kong, SAR. 

## 2. Methodology

This study is comprised of three major components. The performance of the absorbent on NO_2_ removal and the impact of absorbent on gas stream RH were first evaluated. Then the physical humidity conditioning by the Nafion tube was assessed. Finally, a comprehensive field evaluation of the system was carried out in different environments with a mathematical correction algorithm employed.

### 2.1. Test Setup

**Figure 1:**
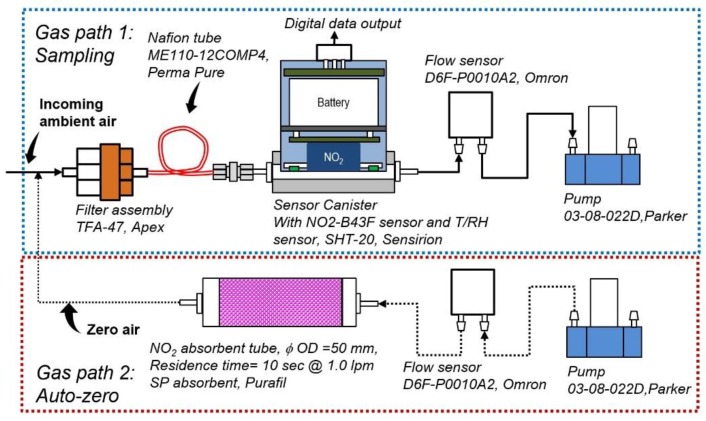
Schematic of the experimental setup.

Figure 1 shows the experimental setup used to generate NO_2_ free air and to condition the humidity of incoming air. There are two alternate gas paths in the setup. One for direct NO_2_ sensor measurement (gas path 1) and the other for NO_2_ free air supply and sensor zeroing (gas path 2). In gas path 1, a pump (03-08-022D, Parker Hannifin Corp., Cleveland, OH, USA) was included, introducing target air samples at 0.5 L/m through (1) a Teflon T-union, (2) a filter assembly (TFA-47, Apex Instruments, Inc., Fuquay Varina, NC, USA) holding a PTFE filter (450-47-4, Savillex Corp., Eden Prairie, MN, USA) with a five-micron pore size and for coarse particle removal, (3) a 30 cm long Nafion tube (ME110-12COMP4, Perma Pure LLC, Lakewood, NJ, USA) with a 2.2 mm inside diameter and a 2.7 mm outside diameter for humidity equilibration of air passing through the tube, (4) a NO_2_ sensor canister, and (5) a flow sensor (D6F-P0010A2, Omron Corporation, Hoffman Estates, IL, USA) for monitoring the air flow. In gas path 2, a separate pump was set up to draw air through a cylinder (*φ* 50mm × 140 mm length) filled with the chemical absorbent (SP, Purafil, Inc., Doraville, GA, USA), providing NO_2_ free air intermittently at 1.0 l/m through the system for sensor zeroing. A PCB board was built and programmed to automatically control the switching of the pump in gas path 2. Data including sensor voltage, temperature, RH, time and pump working status were saved to a SD memory card mounted on the PCB board in a 2 s time resolution. All of these components were assembled in a carrying case with the size of 40 cm length × 29 cm width × 12 cm height. The system was suitable for battery operation. 

The NO_2_ sensor used in this study was an electrochemical NO_2_ sensor with ozone filter to minimize the ozone interference (NO2-B43F, Alphasense, Braintree, UK). Extensive evaluations of this sensor’s performance have been documented in the literature, including our earlier investigations. Cross-interference from other common gas pollutants on the test NO_2_ sensor were not considered important based on prior studies [2,10,13,15,16]. They were not included in the experimental design. We have also documented, in prior studies, the impact of environmental factors and the development of correction algorithms to address the impact with improved sensor data accuracy. In this study, we placed the sensor into an anodized aluminum canister (*φ* 47 mm × 54 mm length) which also included a temperature and humidity sensor (SHT-20, Sensirion, Staefa, Switzerland). A 3.7 V, 500 mAh polymer lithium rechargeable battery (503030, Jinxun, Shenzhen, China) was built into the canister to supply a bias voltage between the electrodes for the sensor stabilization. 

During the tests, the system operation was configured for 2 h continuous sensor sampling via gas path 1, referred to as ‘sampling mode’, hereafter, followed by automatic zero air flushing mode for 20 min via gas path 2, referred to as ‘auto-zero mode’.

### 2.2. NO_2_ Sensor Calibration

The performance of the Alphasense NO2-B43F electrochemical cell has been well tested in earlier work and has shown excellent linearity over a range of 5–250 ppb NO_2_ under laboratory conditions [13,16]. Due to the impact of environmental factors on the sensor response, conventional multiple point calibration solely using dry standard gas is not sufficient to fully characterize the sensor performance in atmospheres with varying temperature and humidity [17]. In this study, sensor calibration and correction algorithm development were performed in the ambient atmosphere with side-by-side comparison using a calibrated reference NO_2_ analyzer (Model 405 nm NO_2_/NO/NOx Monitor, 2B Technologies, Inc., Boulder, CO, USA). During the concurrent measurement period, the auto-zero time length of the system was set as 0 so only gas path 1 worked during the calibration period. Equation (1) was used to fit the inputs from sensor output, temperature and RH [13]:

(1) Conc.=aV+bT+cRH+d

where Conc. is the reference NO_2_ concentration in ppb; V is the voltage output of the NO_2_ sensor in mV; T is the air temperature in °C; RH is the relative humidity in %, while *a*, *b*, *c*, and *d* are the coefficients. 

After sensor equation was established, standard NO_2_ gas with different concentration levels (0, 20, 40, 60, 80, 100, 120, 140 ppb) was generated from a dilution system (T700U, Teledyne, Thousand Oaks, CA, USA) with a zero-air generator (T701H, Teledyne, Thousand Oaks, CA, USA), and introduced to the sensor system. Each concentration step was 30 min long. This verified the validity of the sensor equation obtained from ambient environmental measurements and also to determine the precision of the calibrated sensor system.

### 2.3. Performance Test of NO_2_ Absorbent

The NO_2_ absorbent used in this study is a chemical medium known as SP, in a spherical shape of *φ* 3.175 mm with porous structure. It is made of activated alumina, binders and sodium permanganate and is capable of adsorbing and trapping hydrogen sulfide, sulfur dioxide, nitrogen dioxide, nitric oxide, and formaldehyde by chemisorption. The specifications of SP absorbent are listed in Table S1. SP was housed in a sealed glass tube (*φ* 50 mm × 140 mm length). Comprehensive tests were carried out to evaluate the performance of the SP absorbent, including:

(1) NO_2_ removal efficiency

Standard NO_2_ gas with different concentrations (0, 50, 100, 150, 200, 250, 300, 350, 400 ppb) were generated with each concentration step lasting 25 min. The gas was fed into the 2B 405 analyzer at a flow rate of 1.8 L/m with SP tube inline during the last 15 min of each concentration step.

(2) Long-term efficiency

Considering the needs of long term ambient monitoring with frequent zeroing operations, the efficiency of the SP was tested as a function of time. One SP tube was connected with a reference NO_2_ analyzer (T500U, Teledyne, Thousand Oaks, CA, USA) drawing ambient air at the flow rate of 0.9 L/m, with another NO_2_ analyzer, 2B 405, measuring ambient NO_2_ concentration concurrently. This experiment ran for 28 days continuously to evaluate the working performance of SP during long-term usage.

(3) Impact on gas RH

For ambient measurements, the variation of RH may have large impact on NO_2_ sensor response. It was considered likely that the SP absorbent might impact the humidity of the air passing through it. To assess this topic, two sensor canisters were connected upstream and downstream of the SP tube to determine the effect of the absorbent on the gas RH. Zero gas with RH levels around 35% and 70% was then generated from a humidity generator (OHG Humidity Generator, Owlstone Inc., Norwalk, CT, USA) with a 2 L/m flow rate and passed through the sensors and the SP tube.

(4) Ambient test

The sensor system operated in the laboratory for two days with both modes (sampling mode and auto-zero mode) in service, but with no Nafion tube assembled. This was mainly utilized to assess the humidity effect caused by passing air through the SP in ambient conditions.

### 2.4. Performance Test of Nafion Tube

Nafion is a polymer of tetrafluoroethylene and perfluoro-3,6-dioxa-4-methyl-7-octene-sulfonic acid, possessing the characteristics of Teflon (resistant to the chemical reaction) and sulfonic acid (with very high water-of-hydration). Nafion functions essentially as a highly-selective, semi-permeable membrane to water vapor. Nafion can be formed into a tube and if the gas inside the tube is wetter than the gas outside the tube, drying of the airstream will occur. If the outside gas is wetter, humidification will occur. The drying/wetting efficiency mainly depends on the humidity differences inside and outside the tube. 

The test of Nafion tube mainly focused on three topics: 

(1) NO_2_ loss

Our working assumption is that NO_2_ passes through the tubing without chemical loss. In order to evaluate the impact of Nafion tubing on the NO_2_ loss at ambient level concentration, standard NO_2_ gases with different concentration levels (0, 50, 100, 150, 200 ppb) were generated, then flushed to the reference 2B 405 NO_2_ analyzer. The Nafion tube was connected inline intermittently for each step to determine NO_2_ loss under different concentrations.

(2) Laboratory humidity equilibrium performance

**Figure 2:**
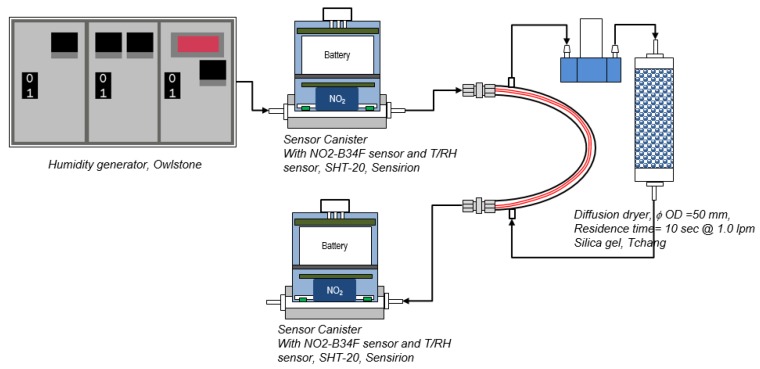
Setup of humidity equilibrium test of Nafion tube with a humidity generator.

A stainless-steel U-tube was fabricated to house the Nafion tube and sealed with 1/4’’ tube fitting nuts at both ends but leaving two connectors of Nafion tube out. As shown in Figure 2, a pump and a diffusion dryer filled with silica gel were connected with two 1/4’’ tubes of the U tube to pump dried gas continuously into U tube, surrounding the Nafion tube with air of desired humidity. Two sensor canisters were installed upstream and downstream of the Nafion tube to measure the change of RH. Zero gas with RH levels of approximately 40%, 60%, 80%, and 90% were generated by the humidity generator and flushed through the Nafion tube, and each step lasted approximately 15 min. After the controlled humidity test, a longer-term test was performed with ambient air drawing through the Nafion tube to validate the humidity equilibrium dynamics.

(3) Humidity equilibrium with gas absorbent

Since the NO_2_ absorbent’s moisture retaining capacity may vary with operation and result in a sudden change of sample air humidity during mode switching, this test included the Nafion tube in the gas line as shown in Figure 1 to investigate its performance in equilibrating the varying humidity of the sample air. The test was carried out in the laboratory for two days with a two-hour cycle of ambient air sampling for 120 min and absorbent line for 20 min, with the Nafion tube in the upstream of the sensor canister. 

### 2.5. Sensor System Field Test

Three urban microenvironments were selected for the field test of the sensor system over a three-month time period. The first test environment is a ventilated indoor office environment controlled by air conditioners, located in Shek Kip Mei, Hong Kong (22°20'00.1"N 114°10'11.5"E). Relatively stable temperature and RH and low NO_2_ concentration were expected in this environment. The second test environment was also an indoor office environment, located in Kowloon Tong, Hong Kong (22°20'08.9"N 114°10'22.7"E), but it is well controlled by the central air-conditioning system. Stable temperature and RH and low NO_2_ concentrations were also expected in this environment. The third one was a sheltered roadside environment along a busy main road, located in Shum Shui Po, Hong Kong (22°19'41.0"N 114°09'41.0"E). An environment with larger NO_2_ concentration variation as well as larger variations in temperature and RH was expected at this site. During the field test, both the developed sensor system, and reference NO_2_ analyzer (2B 405) were set up side-by-side with an additional measurement of ambient temperature and RH. The whole test system including sensor system, references was placed at the chosen environments in concession to identify the robustness of the auto-zero correction for the sensor drift in different environments and in long-term operation. 

## 3. Results and Discussion

### 3.1. NO_2_ Sensor Calibration

**Figure 3:**
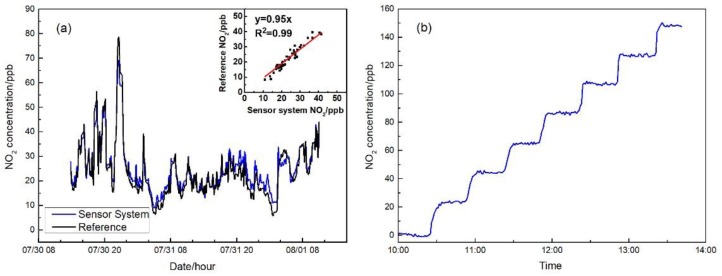
(**a**) Comparison of time series NO_2_ concentrations from the sensor system and the reference analyzer during calibration; and (**b**) standard NO_2_ gas with different concentrations from the dilution system measured by the calibrated sensor system.

During the calibration, a total of 2300 min of data were collected and the one minute data of sensor voltage (in mV), temperature (in °C), RH (in %) were fit into Equation (1) with reference NO_2_ data (in ppb) to derive the coefficients *a*, *b*, *c*, and *d* by multiple linear regression, which were obtained as 6, 4, −0.43, and −165, respectively. With 95% confidence, *p*-values of coefficients *a*, *b*, *c*, and *d* were all less than 0.05 and standard errors of each coefficient were 0.07, 0.4, 0.1, and 10. Figure 3a shows the comparison of the reference and the sensor system data after correction. Those two datasets have good agreement in the concentration range from near 0 to 80 ppb. The inset shows the scatterplot of the two datasets in one hour averages with good linearity and a 0.99 *R*^2^. In Figure 3b, the response of the sensor system to the standard gas in the range of 0–140 ppb is displayed after correction, which indicates a 2 ppb precision of the sensor system and high linearity (0.94) of the sensor response to the target gas, with a 0.99 R^2^. 

### 3.2. NO_2_ Absorbent Performance

**Figure 4:**
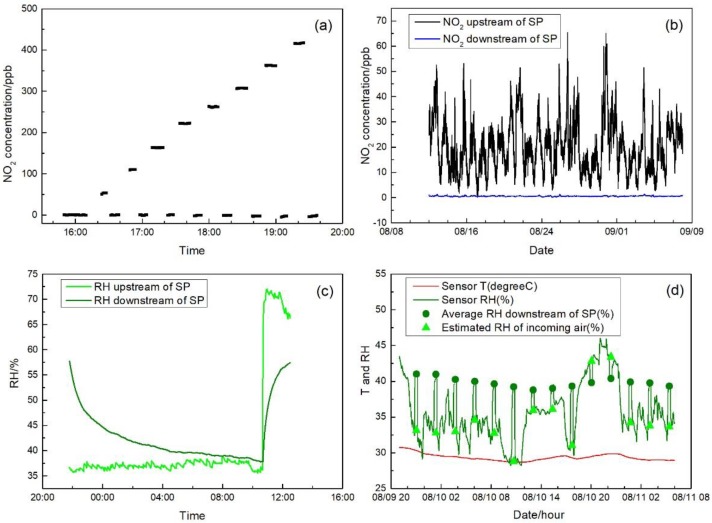
(**a**) Standard NO_2_ gas with different concentrations from dilution system measured by the 2B 405 with and without the SP tube; (**b**) NO_2_ concentrations measured upstream and downstream of the absorbent in a long-term measurement; (**c**) RH measured upstream and downstream of the SP tube with 35% and 70% RH inputs; and (**d**) RH and temperature measured during the sensor ambient test. RH downstream of SP during auto-zero mode is averaged and displayed as dots. RH estimated by those before and after auto-zero are also displayed as triangles.

Figure 4a shows the NO_2_ removal performance of the tested absorbent at different dry NO_2_ concentrations. For all the steps from 0 to 400 ppb, the absorbent showed consistent efficiency in reducing NO_2_ to near zero concentration. Regarding the long-term performance and reduction capacity, Figure 4b presents the NO_2_ concentration upstream and downstream of the absorbent following the 28-day test with continuous incoming ambient air and varying NO_2_ concentration up to 70 ppb. The NO_2_ concentration downstream of the absorbent was well controlled to approximately 0.6 ± 0.2 ppb showing the feasibility of the absorbent system usage for long term deployment in ambient monitoring. 

In order for the sensor output to properly respond to the removal of NO_2_, RH of air is also an important factor to address according to our earlier investigations [13]. Figure 4c shows the impact of the absorbent on the RH of incoming air. Initially, the absorbent tube was equilibrated in air with RH of 60%. When the zero air with a steady 35% RH passed through the absorbent tube, the air RH decreased slowly from initial 60% and it took about nine hours to reach the humidity equilibration matching with the incoming condition. Following the equilibration, the input moist zero air was switched to RH of 70% and the output air RH gradually increased to catch up with the change of incoming air condition. For both humidity test sections, during the first 20 min after switching the incoming air condition, the RH downstream of the absorbent had very limited range of alternation within 5%. Therefore, in the practical application, when switching between auto-zeroing and sampling, the absorbent has a predictable humidity impact regardless of the incoming ambient conditions. 

Figure 4d shows the humidity effect of the absorbent during ambient air sampling test. The green line shows the RH of the sample air through the NO_2_ sensor canister during ambient sampling and auto-zeroing periods. The triangles represent the estimated RH of ambient air during auto-zeroing, determined by the linear interpolation of RH before and after auto-zeroing. The dots are the RH of the air after passing through the absorbent tube during the auto-zero period. During the test, the ambient incoming air temperature was relatively stable at around 28 °C to 31 °C; however, the ambient RH had a wide range from 30% to 50%. Over the 33-h continuous test period, the average RH during zero mode in the downstream of the absorbent was stable within a narrow range of 39% to 41%, despite the wider variation of incoming air condition. Sharp RH changes were observed as indicated by the triangles and dots during the switching due to the capacity of the absorbent in retaining moisture. No temperature change was induced by the absorbent. Under very low concentration conditions, such RH changes may be a challenge to the sensor response to NO_2_, making the current zero process impractical. Further discussion will be given in Section 3.4.

### 3.3. Nafion Tube Performance

**Figure 5:**
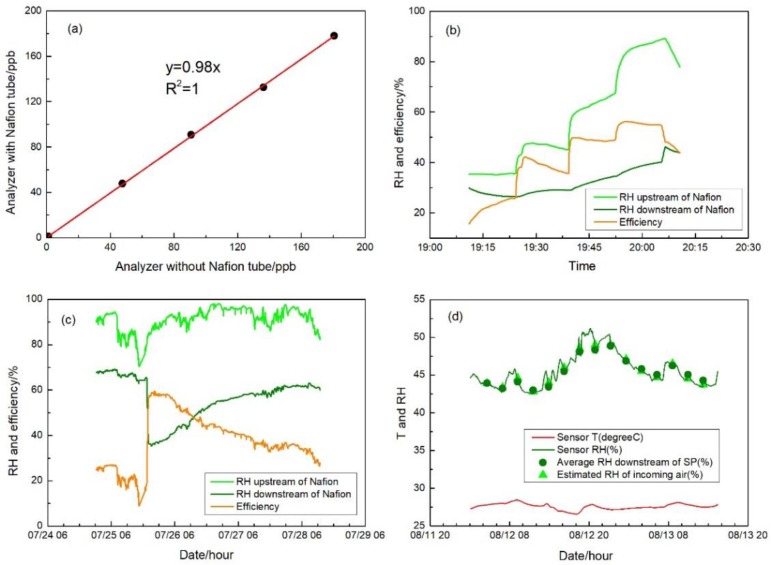
(**a**) Scatterplot of readings from the NO_2_ analyzer with the Nafion tube vs. without the Nafion tube; (**b**) short-term humidity equilibrium test of the Nafion tube using the humidity generator; (**c**) long-term humidity equilibrium test of the Nafion tube using ambient air; and (**d**) RH and temperature measured during the gas absorbent test with Nafion tube inline. RH downstream of SP during auto-zero mode is averaged and displayed as dots. RH estimated by those before and after auto-zero is also displayed as triangles.

Figure 5a presents the comparison of the NO_2_ concentrations at different steps upstream and downstream of the Nafion tube. There is no discernable difference of concentrations in the range of 0–180 ppb with slope of 0.98 and R^2^ of 1, as shown in the figure, demonstrating there is no NO_2_ loss using the Nafion tube inline. The Nafion tube sealed in the air-tight U tube serves as a buffer of changing humidity conditions for incoming air. Figure 5b shows the comparison of RH upstream and downstream of the Nafion tube during the short-term efficiency test. Although the RH of incoming air spanned from 40% to 90% in multiple steps, the Nafion tube stabilized the RH within a narrower range from 30% to 50%. The fast transition between RH steps with an average of 20% gap within 1 min had no immediate impact on the downstream RH demonstrating the effective humidity buffering effect of the Nafion tube in lessening the impacts of humidity in short term operation. The results also support the potential applicability of the Nafion tube for sensor-based exposure assessment in which the fast change of microenvironments with the sudden change of air condition is expected. In the follow-up long-term operation test shown in Figure 5c, recirculating dry air was injected during the test to investigate the effectiveness of such operation by days. When there was high incoming air RH at around 80–90%, the Nafion tube was first balanced at around 60–70% RH. Immediately after the dry air injection was on, the RH downstream of the Nafion tube was lowered to 40%, but slowly climbed to 50% after 20 h operation, and further to 60% after 50 h. Following the short- and long-term Nafion tube test, we integrated the Nafion tube with the auto-zero module upstream of the NO_2_ sensor, as shown in Figure 1. Figure 5d shows the air temperature and RH with the switching flow between ambient sampling air and auto-zero air. Different from Figure 4d, the introduction of the Nafion tube clearly stabilized the RH, even during the auto-zero period with the elimination of the sudden shift of RH. This set of experiments demonstrates the Nafion tube installed inline of the air flow can effectively buffer the sudden change of the incoming air RH, and also equilibrate the rather large variations into a narrowed range. 

### 3.4. Impact of Humidity Stabilization on Sensor Output

**Figure 6:**
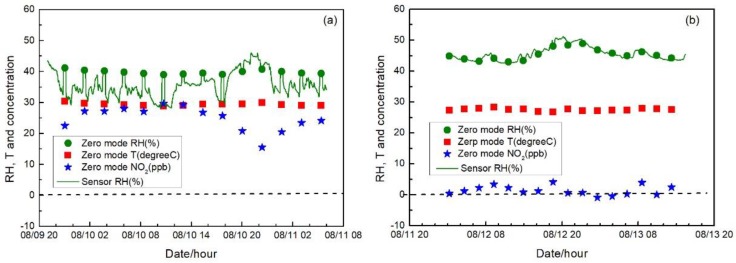
(**a**) Time series of RH measured by sensor system during the absorbent ambient test, and RH (green dots), temperature (red squares), and calculated NO_2_ concentration (blue stars) during auto-zero modes; (**b**) time series of RH measured by sensor system during the ambient test with Nafion tube inline, and RH (green dots), temperature (red squares), and calculated NO_2_ concentration (blue stars) during auto-zero modes. A dashed dark line is displayed indicating the zero NO_2_ concentration.

During the ambient test on the absorbent impact on sample RH and Nafion test on humidity stabilization, we have shown the NO_2_ free air passing through the absorbent induced a sudden change of RH (Figure 4d) and the introduction of Nafion tube inline was effective in stabilizing the RH (Figure 5d). Figure 6 shows the comparison of impact of RH change with and without Nafion humidity stabilization on the accuracy of sensor output. During the absorbent ambient test, the air temperature was relatively stable around 30 °C while operating in auto-zero mode. Meanwhile, the presence of the NO_2_ removal absorbent produced a stable RH output at around 40%. However, a sudden change of RH occurred while switching between ambient air and NO_2_ free air. This caused a large deviation of the sensor output concentration from zero as shown in Figure 6a. The calculated NO_2_ concentration during auto-zero mode ranged between 15–30 ppb. One possible reason is that the gas sensor responds to the sudden and large change of sample air RH having a long-tailed lag to allow the stabilization of sensor electrolyte for normal and accurate response to zero concentration air. In contrast, the introduction of Nafion tube inline between the absorbent and the NO_2_ sensor reduced this markedly, as shown in Figure 6b. While the incoming air temperature was still relatively stable around 30 °C during auto-zeroing, RH had a variation ranging from 40% to 50%, but there was no sudden change of RH during mode switching due to the effective humidity stabilization performance of the Nafion tube. Consequently, the calculated NO_2_ concentration during auto-zero mode was observed centering around zero in the narrow range of −2 to 5 ppb during the two-day test, demonstrating the importance of the humidity stabilization on the sensor output during auto-zero operation. 

### 3.5. Sensor System Field Validation

**Figure 7:**
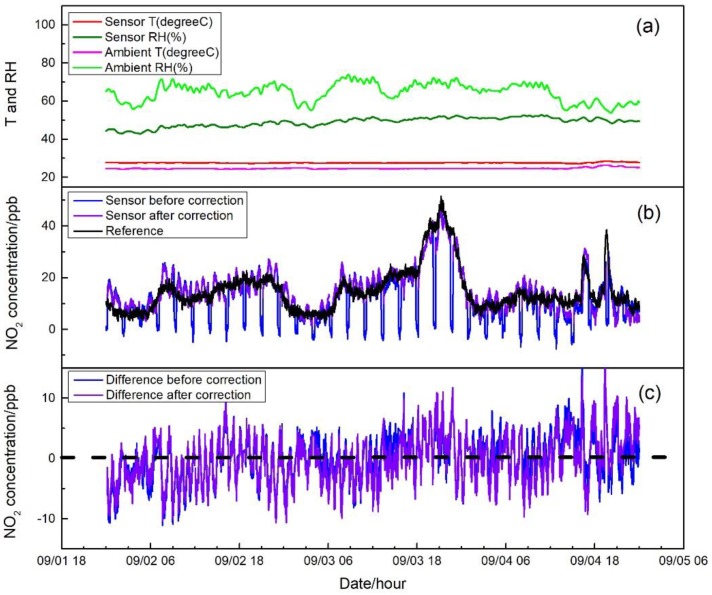
(**a**) Time series of temperature and RH of incoming ambient air and sensor system during the ventilated indoor test; (**b**) NO_2_ concentration measured by the sensor before and after the zero correction and by the reference instrument. Concentrations of auto-zero modes are displayed in the dataset of the sensor concentration before correction; and (**c**) NO_2_ concentration difference between the sensor system and the reference before and after zero correction.

**Figure 8:**
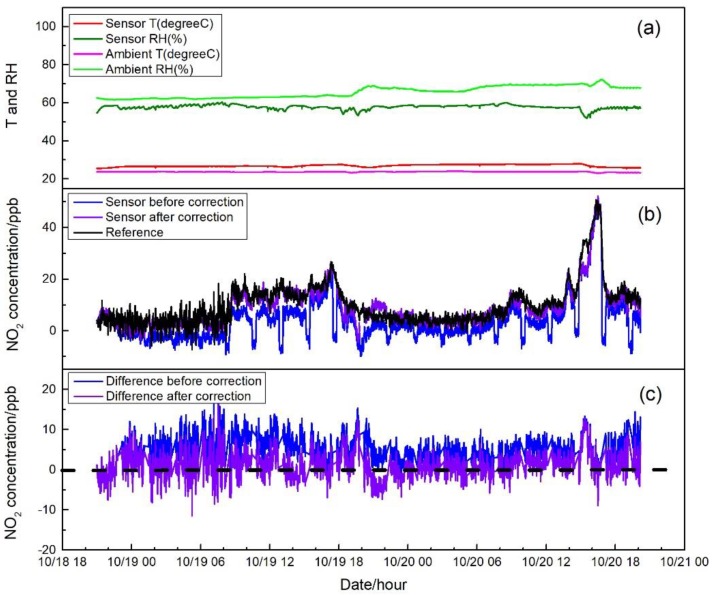
(**a**) Time series of temperature and RH of incoming ambient air and sensor system during the central air-conditioned indoor test; (**b**) NO_2_ concentration measured by sensor before and after the zero correction and by the reference instrument. Concentrations of auto-zero modes are displayed in the dataset of sensor concentration before correction; and (**c**) NO_2_ concentration difference between the sensor system and the reference before and after zero correction.

**Figure 9:**
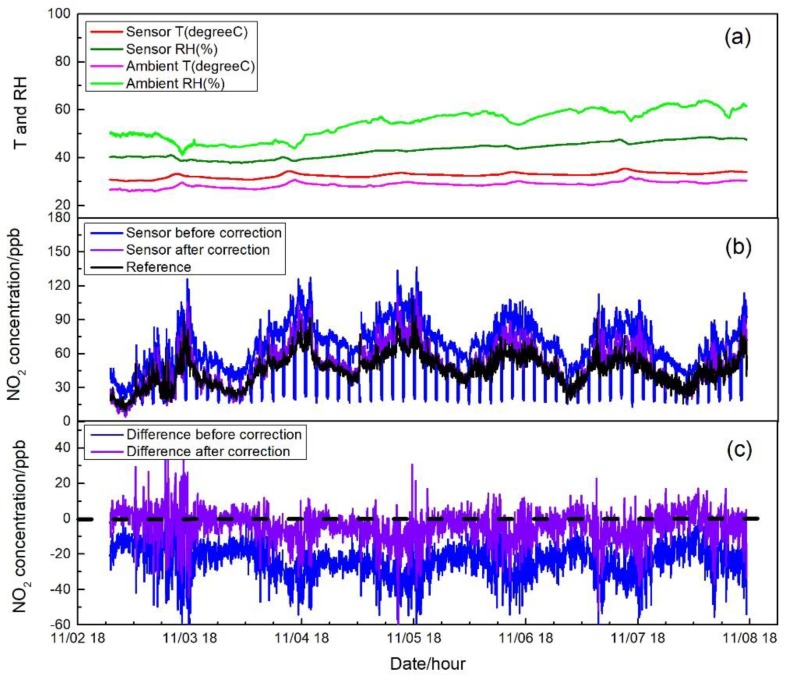
(**a**) Time series of temperature and RH of incoming ambient air and sensor system during a roadside environment test; (**b**) NO_2_ concentration measured by the sensor before and after the zero correction and by the reference instrument. Concentrations of auto-zero modes are displayed in the dataset of the sensor concentration before correction; and (**c**) NO_2_ concentration difference between the sensor system and the reference before and after zero correction.

Figure 7, Figure 8 and Figure 9 show the measurement results in three different environments, including sensor raw and zero air-corrected NO_2_ concentration, reference NO_2_ concentration, ambient air conditions, and sensor temperature and RH. Zero air correction of NO_2_ concentration was done by averaging the raw concentration of the last 15 min of each 20-min zeroing data to calculate the concentration offset from zero, followed by applying the offset to the following sampling period.

Figure 7a shows the 1-min average data of the sensor system field test in a ventilated indoor environment including sampling and auto-zero modes. Temperatures measured outside or inside the sensor system were relatively stable around 25 °C and 27 °C, respectively. The measured ambient RH ranged from 50% to 75%. However, with the Nafion tube inline, the sample air was lowered to a much narrower range of 40–50% and no sharp RH change was detected, even during auto-zero modes. Ambient NO_2_ ranged from 5 to 50 ppb with a mean of 16 ppb. Auto-zero data were only displayed in the raw data of sensor before zero correction in Figure 7b. The measured concentrations during the auto-zero periods were −4 to 4 ppb with a mean of zero. Further, the NO_2_ concentration difference between the sensor system and the reference analyzer was calculated and demonstrated in Figure 7c with a dashed dark line representing zero. The average and the standard deviation of the difference before zero correction were 0.1 ppb and 3.9 ppb, and 0.4 ppb and 3.9 ppb after the correction. The median and median absolute deviation were 0.02 ppb and 2.7 ppb before zero correction, and 0.52 ppb and 2.7 ppb after the correction. No apparent difference was observed among the NO_2_ concentration from the sensor system and the reference analyzer before or after zero correction, showing the stable sensor performance.

In tests in the central air-conditioned indoor environment and the roadside environment, similar variation patterns of temperature and RH were observed with that in the first field test. As shown in Figure 8a and Figure 9a, temperatures measured outside or inside the sensor system in the central air-conditioned indoor environment and the roadside environment were all relatively stable. For RH, whether in the air-conditioned indoor environment (60% to 70% RH) or in the roadside environment (40% to 60% RH), ranges were narrowed and buffered by the Nafion tube without abrupt change even when zeroing. It can be seen in Figure 8b and Figure 9b that when the system was operated in different microenvironments with different ambient conditions, the sensor raw NO_2_ concentration followed similar trends to those from the reference analyzer; however, there were differences between the outputs of these two sampling systems. After the sensor concentration was corrected by auto-zero, the differences were mitigated and good agreement was shown among the sensor system and the reference. The differences were calculated and shown in Figure 8c and Figure 9c for the two test environments. It can be seen that the concentration differences after sensor correction varied more around zero compared to that before correction. In Figure 9c, when the ambient NO_2_ concentration was relative high, approximately around the hundred ppb level, an average difference around 10 ppb still existed even after zero correction. There were two factors that may contribute to these differences. First, in the electrochemical sensor, there are four electrodes, called the working electrode, the reference electrode, the counter electrode, and the auxiliary electrode. The chemical reaction (reduction or oxidation) of the target gas occurs at the working electrode, which generates an electron flow between the working electrode and counter electrode [2]. The sensor response mainly depends on the diffusion of the reaction gas to the working electrode [18]. To eliminate the cross-interference from ozone, a chemical filter is added above the working electrode [19] and may have an impact on the response of the sensor under high concentration condition. Second, the outdoor environment measured had complex gaseous pollutants related to the impacts of traffic, in which cross-sensitivity of test NO_2_ sensor may exit and induce the observed difference. The average and standard deviation of the NO_2_ concentration difference between sensor system and reference monitor before and after correction were 5.0 ± 3.4 ppb and 1.1 ± 3.2 ppb for the central air-conditioned indoor environment; −23.7 ± 9.0 ppb and −4.0 ± 8.0 ppb for roadside environment. The median and median absolute deviation before and after correction were 5.0 ± 2.0 ppb and 1.0 ± 1.9 ppb for the central air-conditioned indoor environment; and −23.1 ± 5.3 ppb and −3.3 ± 4.6 ppb for the roadside environment. Overall, during the application of the sensor system, drift of sensor response to the target gas was observed due to the environmental change and the long-term operation of the system prior to the inclusion of the SP absorbent, which produced an offset of the original equation for sensor concentration calculation compared with references. The auto-zero functioned as expected to compensate the induced drift. 

## 4. Conclusion

Low-cost sensors and sensor-based air monitoring systems have received considerable attention. Electrochemical sensors, as one type of sensors for gaseous pollutants, have been widely employed for air quality monitoring because of their low cost, easy deployment, low energy consumption and small size. However, the effective use of these sensors for air monitoring is not without problems. Their drawbacks cannot be ignored during their application in various microenvironments. Electrochemical sensors may respond differently to a given concentration of their target gas under varying ambient conditions, for example, the presence of interfering gases, and variations in temperature and humidity can influence sensor response, which would contribute to inaccurate air monitoring results. Further, the long-term stability of these sensors is in question with little data currently available on this issue.

Following the traditional air monitoring protocol, this study has designed an effective modification to an electrochemical sensor based system for field monitoring of ppb level NO_2_. An automated sensor zeroing protocol was developed and tested using a chemical absorbent to remove NO_2_ as a means to perform automatic zero correction for NO_2_ sensors in ambient urban air of Hong Kong. This study evaluated the removal performance of absorbent under different conditions and RH variation. Good NO_2_ removal performance was observed both in short-term operation with standard gas of different concentrations and in long-term operation under ambient condition. It was also found to bring the impacts of RH change on the sensor reported results. A Nafion tube was further introduced and its performance was assessed as a means to stabilize the impacts of sharp RH changes. The robustness of drift correction by auto-zero were estimated successively in different microenvironments. Ambient temperatures in the test environments were relatively stable but the RH changed in a broad range from 40% to 75%. When the system was moved to different test environments, concentration drift was observed, however, zero correction mitigated it effectively. After the correction, NO_2_ concentration from the sensor fit well with that from the reference analyzer. The humidity equilibrium and auto zero protocol narrowed the difference between the sensor system and the reference, and significantly improved the performance of the electrochemical sensor for long-term unattended operation.

In this study, the system was shown to be effective with zeroing every 2 h. This frequency of zeroing may possibly be reduced without a major cost in data quality, however, further experimentation would be needed to determine if a lesser frequency is adequate. It should be noted that because this protocol is automated and appears robust, there is little cost by the current frequent zeroing practice. Further, this demonstration was only done with one of the common air pollutants. We are working to evaluate whether similar protocols might improve data quality for these pollutants. This sensor system was only tested in relatively stable environments that included a limited range of conditions. It did not include the consideration of temperature impacts on the electrochemical sensors. These have been documented to some extent, however, it remains to be determined whether the sensor system performance would be improved if temperature parameters were controlled. Technical approaches for temperature control could be considered to stabilize the sample air temperature, which will be evaluated in subsequent tests.

## Supplementary Materials

The following are available online at www.mdpi.com/1424-8220/17/8/1916/s1, Table S1: Specifications of SP.
