# Supplementary material for: Development and Evaluation of A Novel and Cost-Effective Approach for Low-Cost NO2 Sensor Drift Correction
Source: Sensors (Basel). 2017 Aug 19;17(8):1916. doi: 10.3390/s17081916 (PMC5580082; doi:10.3390/s17081916)
Supplement: Supplementary file 1 [file sensors-17-01916-s001.pdf]

Table S1. Specifications of SP.

Sodium Permanganate	Moisture	Bulk Density	NO_2 Removal Capacity
12% (min) as $NaMnO_4$	35% (max)	0.8 g/cc ± 5%	0.22 g/cm^3
